# Unveiling the hidden struggles: Exploring the profound impact of advanced stage cutaneous T‐cell lymphoma on quality of life

**DOI:** 10.1002/ski2.300

**Published:** 2023-10-26

**Authors:** Rosanne Ottevanger, Judith S. Feenstra, Liesbeth M. van Vliet, Sylvia van Beugen, Andrea W. M. Evers, Cees Kennedy, Rein Willemze, Maarten H. Vermeer, Koen D. Quint

**Affiliations:** ^1^ Department of Dermatology Leiden University Medical Center Leiden The Netherlands; ^2^ Institute of Psychology, Health, Medical and Neuropsychology Unit Leiden University Leiden The Netherlands

## Abstract

Erythrodermic mycosis fungoides and Sézary syndrome are chronic, relapsing‐remitting diseases that greatly impacts patients' quality of life (QoL). *Mogamulizumab‐kpkc* (Mogamulizumab) is a novel therapeutic agent for cutaneous T‐cell lymphomas with a notable impact on progression‐free survival. Qualitative assessment methods allow a broader exploration and greater insight in individual patient experience than quantitative studies. However, there is limited data on the impact of mogamulizumab on health‐related QoL. To investigate the impact of erythrodermic cutaneous T‐cell lymphoma (E‐CTCL) on QoL and the effect of mogamulizumab on the QoL. Semi‐structured interview were conducted with seven patients with E‐CTCL that were receiving mogamulizumab treatment. Five major themes arose: Diagnosis and the diagnostic delay and uncertainty experienced by participants; Physical functioning due to the high symptom burden; Psychological and social functioning considering the significant impact on daily life; Treatment and the effect of mogamulizumab; and Support by family, friends and health professionals. Mogamulizumab therapy resulted in a significant decrease of symptoms. The small sample size should also be taken into account although data saturation was reached. This study gives a broad insight into the large impact of E‐CTCL and the major consequences on the physical functioning as well as on the emotional/psychological and social well‐being. Mogamulizumab appears to have a positive effect on symptoms.

**What is already known about this topic?**

- Exploring the impact cutaneous T‐cell lymphoma on quality of life by qualitative assessments allow a broader exploration and great insight in patient experience.

**What does this study add?**

- To our knowledge, this study is the first qualitative assessment that studies the effect of mogamulizumab and the impact this treatment has on patients' quality of life. The findings from this study provide valuable information to healthcare professionals and guide them in improving their care strategy and provide realistic expectations.

## 1 INTRODUCTION

Cutaneous T‐cell lymphomas (CTCL) are a rare and heterogenous group of non‐Hodgkin lymphomas. Mycosis fungoides (MF) and Sézary syndrome (SS) are the classic types of CTCL. MF is the most common type of CTCL and follows a chronic slowly progressive disease course. It classically presents with erythematous patches and plaques that can progress into tumours and/or erythroderma, with or without extra‐cutaneous involvement.^1^ SS, a less frequent leukaemic variant, is defined by the triad of erythroderma, generalised lymphadenopathy and blood involvement.^1^, ^2^ Early‐stage MF carries a highly favourable prognosis, whereas advanced‐stage MF and SS patients have a much lower median survival.^3^

However, CTCL has a heterogenous phenotype and therefore can cause a broad spectrum of symptoms significantly impacting patients' quality of life (QoL).^4^ In general, erythrodermic CTCL (E‐CTCL) patients, in which patients with MF or SS present with a complete red skin and report a lower QoL than patients with early stage disease.^2^ QoL measurements in CTCL patients is often measured with standardised validated questionnaires and reported on multiple domains.^2^ However, quantitative tools in these studies seem insufficient in depicting all aspects that are of importance for QoL in CTCL patients.

As CTCL generally has a chronic symptomatic course and since there are scarce curative treatment options, the primary focus of treatment is to minimise symptoms, whilst at the same time reducing the risk of disease progression.^1^, ^5^ The limited treatment options for E‐CTCL patients often concern intensive therapies while positive effects are mostly short‐lasting with high rates of adverse effects. Recently, *Mogamulizumab‐kpkc* (Mogamulizumab), a humanised monoclonal antibody, was approved for the treatment of relapsed or refractory MF and SS.^6^ This novel therapeutic agent exhibited a good response on symptoms and has shown to improve patients' QoL.^7^, ^8^ Qualitative assessment methods allow a broader exploration and greater insight in patient experience.^9^ There are few quantitative studies assessing QoL that purely focus on patients with E‐CTCL.^2^ In addition, to our knowledge no qualitative studies in advanced stage CTCL has been conducted with patients receiving Mogamulizumab. This qualitative study aims to further investigate the impact of erythrodermic MF and SS on patients' QoL and to explore the effect of mogamulizumab on the QoL.

## 2 METHODS

### 2.1 Study design

Semi structured in‐depth interviews were conducted in patients with erythrodermic MF or SS (E‐CTCL) that were receiving mogamulizumab. The study design and results are reported according to the consolidated criteria for reporting qualitative research (COREQ).^10^ The study design was approved by the Medical Research Ethics Committee Leiden Den Haag Delft, number N22.002. Written informed consent was obtained from each participant.

### 2.2 Setting

The qualitative study was conducted at Leiden University Medical Center, the national referral centre for cutaneous lymphomas in the Netherlands. Diagnosis of cutaneous lymphomas are made through clinicopathological correlation by the Dutch Cutaneous Lymphoma group according to the WHO‐EORTC classification.^1^

### 2.3 Recruitment

Patients with a known diagnosis of E‐CTCL that were being treated with mogamulizumab infusions between March 2022 and June 2022 were eligible to participate in this study. Staging was confirmed by providers. Eligible patients were recruited by their consultants through purposive sampling. All patients that were eligible agreed to participate, resulting in a 100% participation rate.

### 2.4 Data collection

All in‐depth interviews were conducted by the same, single investigator using a semi‐structured interview guide. The interviews were audio recorded and additional field notes were made for each participant subsequent to the interview. *Visual Analogue Scale (VAS) for itch, pain and fatigue was administered to all participants on the day of the interview*. Interviews were conducted until no additional themes or new insights were identified and data saturation was achieved.

### 2.5 Analysis

All interviews were transcribed verbatim, anonymised and imported into the Atlas.ti qualitative data analysis software. The thematic analysis approach was used to analyse the collected data.^11^ Two researchers analysed the data independently prior to comparing the coded transcripts. Firstly, both researchers familiarised themselves with the collected data and after this the initial codes were produced. Further analysis of the coded data generated broader themes and all codes were sorted into the corresponding themes.

## 3 RESULTS

Seven patients participated in this study after which data saturation was achieved. The participants' characteristics are described in Table 1. Six participants had SS and one patient erythrodermic MF, all with disease stage IVa. Patients had a mean age of 67 years (range 49–86). The mean time since diagnosis was 51 months and the number of mogamulizumab infusions ranged between 8 and 34 infusions. The interviews had an average duration of 56 min. Data analysis generated five major themes: diagnosis, physical functioning, psychological and social functioning, treatment and support. Quotations illustrating the major themes are portrayed in Table 2 and cross‐referenced in the text by their quotation number.

**TABLE 1:** The participants' characteristics.

Patient no.	Age (years)/sex	Diagnosis	WHO‐EORTC stage	Time since diagnosis (months)	Prior skin directed therapies	Prior systemic therapies	CCI score	Fitzpatrick skin type	No. of mogamulizumab infusions	CD4/CD8 ratio at start of treatment	CD4/CD8 ratio at time of interview	VAS itch	VAS pain	VAS fatigue
1	65/F	SS	IVA	66	Topical corticosteroids	PUVA, prednisone	0	2	34	69	0.64	0	0	7
2	49/M	MF	IVA	99	Topical corticosteroids	MTX, prednisone, leukeran	1	5	25	26.92	0.76	0	0	0
3	86/F	SS	IVA	62	Topical corticosteroids	MTX, prednisone	3	2	8	4.06	2.65	7	0	4
4	61/M	SS	IVA	28	Topical corticosteroids, UVB	Acitretine, prednisone	2	2	26	6.29	4.11	0	0	6
5	68/M	SS	IVA	68	Topical corticosteroids, UVB	PUVA, prednisone	0	2	9	77.79	4.99	1	1	5
6	59/F	SS	IVA	8	Topical corticosteroids, UVB	MTX, prednisone	0	2	9	113.88	1.37	4	0	3
7	63/F	SS	IVA	25	Topical corticosteroids, UVB	MTX, PUVA, prednisone	0	2	32	10.95	16.68	5	1	6

Abbreviations: CCI, Charlson comorbidity index; MF, mycosis fungoides; MTX, methotrexate; PUVA, psoralen and ultraviolet A; SS, Sezary syndrome; UVB, ultraviolet B; VAS, Visual Analogue Scale.

**TABLE 2:** Quotes.

Theme	Quote
Diagnosis	
Q1	‘And uhh, yeah, then it took a really long time, and don't even ask me… but we're definitely talking years rather than months.’
Q2	‘And it's really still totally surreal… like yeah… What's on earth's happening to me? Yeah, like I'm stuck in a nightmare.’
Q3	‘For me, strange as it may sound, it was a relief. You actually hear it quite often with patients with um… Cutaneous T‐cell lymphoma… that it’s a relief when they finally get the diagnosis because it's taken so long to get there.’
Physical functioning	
Q4	‘I couldn't sleep anymore because of the itching—it drove me completely crazy. At night I would lean against the bathroom wall, naked. Cold water didn't help, cold cream didn't help. It drove me crazy…’
Q5	‘At some points, when the itching was so extreme. I would think Jesus… I can't live like this.’
Q6	‘I could hardly touch my grandchildren because it was just, well… you have really rough hands. So my hands were complete… my skin was complete sandpaper.’
Q7	‘I do notice that sometimes I am completely exhausted—just really, really tyred. Is it because of the moga? Or because of the disease? Or is it just that I'm getting on a bit? A combination of the three, I guess.’
Pscychological and social functioning	
Coping	
Q8	‘And right from the start, my view was “I have a disease, I have to deal with it… uhh… but I am not my illness.’’
Q9	‘It seems as if it hasn't really sunk in yet or something, or maybe it’s also me still keeping it at a distance a bit.’
Q10	‘I also didn't tell people for a very long time. Because I basically didn't want to talk about it.’
Insecurities and awareness/understanding	
Q11	‘And uhm, I just want to go on looking fairly decent. That was already very difficult with the redness, and you can camouflage that a bit, but it's not always possible. (…) yeah, because that just makes me really insecure, you know? I do think it's important to look good. [laughs] So yeah, that's something I find… well, that's difficult, yeah.’
Q12	‘So I'm not going to hide myself away. I wear a bikini on the beach, uhm… even if you see that my skin's bad.’
Q13	‘“You should watch out, your legs are so sunburned.” Everybody has something to say about it.’
Q14	‘Maybe they thought it was catching or something.’
Impact on daily functioning	
Q15	‘Uhm, yeah. I do notice that I sometimes find it more difficult to undertake social activities. Yeah, because I somehow just feel I look different.’
Q16	‘And I see now that those positions have been filled by other people, so yeah, the disease has cut off my career, abruptly cut it off.’
Q17	‘Uhm, I always had to protect my hands, and that meant always wearing gloves, so I had cotton gloves on for the sores and ointments, and then I bought shiny satin gloves to go with my clothes.’
Q18	‘And the skin on my feet was cracked, was constantly cracking open, so the only footwear I could bear was Uggs, because they have a really thick layer of fur, on the bottom too.’
Treatment	
Q19	‘That medication, that did improve my life.’
Q20	‘So I really did go from hell to heaven, in the end.’
Q21	‘And as I said, I'm here now, and at first I went once a week and now once a fortnight. And I feel much better: I'm able to sleep again for a few hours at a stretch.’
Support	
Q22	‘The more time goes by, the more grateful I am to my husband. Because whether my skin looks good or whether it doesn't—it makes no difference to him. And I've learnt to appreciate him much more.’
Q23	‘To start off with I lived next to my sister, so she always put on the ointment at that time because it always had to be done from head to toe.’
Q24	‘I have to say I also have my faith. And it's not like all day every day I… no… For me. And that has given me strength.’

### 3.1 Impact of disease on QoL

#### 3.1.1 Diagnosis

Diagnostic delay was a main topic when discussing the diagnostic process. The majority of the participants experienced a lengthy delay in referral. One patient received the CTCL diagnosis promptly. All other patients experienced a prolonged time to diagnosis, varying between two to 10 years (Table 2; Q1). Eczema was the most common first diagnosis and three patients received treatment for this for multiple years prior to another diagnosis being considered. Uncertainty about the diagnosis and lack of prompt referral often led to dissatisfaction amongst the participants. Three of the participants requested a second opinion in an academic hospital, in all cases this led to their SS/MF diagnosis.

Receiving the diagnosis had an extensive impact on the participants and their families. Many were shocked, upset and/or experienced disbelief (Table 2; Q2). Three patients mentioned that the information about SS on the internet and the associated survival rate contributed to their shock. One patient experienced the diagnosis as a relief considering the diagnostic delay and the associated uncertainty (Table 2; Q3).

#### 3.1.2 Physical functioning

Pruritus was the most prevalent physical symptom (7/7 patients), leading to difficulty in sleeping for four patients. Participants describe the itching to be ‘extreme’ and to ‘drive them crazy’ (Table 2; Q4, Q5). Other reported main symptoms were redness and scaling of the skin. With two patients the severe scaling and cracking of the skin of their hands and feet also resulted in a considerable amount of pain with one participant even refraining from holding her grandchildren due to the roughness of her hands (Table 2; Q6). All patients experienced frequent fatigue and reported that it was caused/increased by the mogamulizumab treatment (Table 2; Q7).

#### 3.1.3 Psychological and social functioning

##### Coping

Patients portrayed various coping mechanisms upon receiving the diagnosis and during their disease. The majority of patients seek to remain positive and to maintain their daily life activities with friends and family. Two participants also mentioned the importance of accepting that they have a disease and not letting it take control of their life (Table 2; Q8). Keeping the diagnosis and its consequences at a distance was another coping strategy for some patients (Table 2; Q9). One patient avoided telling family, friends and colleagues about the disease in order not to have to discuss it. Another patient refrained from searching for information online (Table 2; Q10). Fear amongst patients was mainly caused by uncertainty about the future and disease course.

##### Insecurities and understanding

The visibility of SS/MF is an important cause for insecurities or embarrassment amongst patients. One of the participants mentioned that her appearance is of great importance to her and that the visible symptoms cause feelings of insecurity (Table 2; Q11). Two patients reported avoiding social activities due to these insecurities. Four participants expressed that they would not let the visibility of CTCL cause them to feel ashamed or to cover up (Table 2; Q12).

The visible symptoms of redness and/or scaling of the skin also leads to judgement and unsolicited remarks or advice. Two patients discussed regularly being told to be aware of the sun and their sunburnt skin (Table 2; Q13) and one patient noticed that people would avoid him as they thought he was potentially contagious (Q14) Embarrassment and judgement were the main motivators for participants to avoid social activities or to cover their skin.

##### Impact on daily functioning

The CTCL diagnosis has a significant impact on the daily functioning of patients. As previously mentioned, it causes some patients to refrain from social interactions (Table 2; Q15), however it also has a large effect on career, hobbies, and clothing options.

The majority of patients (5/7) mentioned that the disease had an impact on their ability to work. These participants are now partially or fully on sick leave and some have decided to shift their focus from their career to other things such as family and friends since receiving the CTCL diagnosis. One patient says she finds it difficult that she is not able to continue her work as she still had the ambition and opportunity to further climb the career ladder (Table 2; Q16).

The consequences of MF and SS also impacted certain (sportive) hobbies of the participants and patients were unable to continue to the same potential as before their diagnosis. The main cause for this was fatigue and decreased physical stamina.

A main theme that arose when discussing impact of CTCL on daily life were the clothing adjustments. In addition to covering the skin, the physical symptoms were another main reason for patients to make clothing adjustments. Two patients regularly wore gloves due to the scaling and bleeding of their hands and the associated pain, with one of these patients even having to sleep with gloves on (Table 2; Q17). The extreme dryness and cracking of the skin also led to a participant exclusively being able to endure footwear with a fur bottom (Table 2; Q18). Certain fabrics or garments such as jeans and bras were mentioned to cause additional skin irritation or itching.

### 3.2 Impact of treatment with mogamulizumab

The majority of patients (6/7) were satisfied with the effect of mogamulizumab on their skin and quality of life (Table 2; Q19, Q20). Mogamulizumab infusions resulted in a significant decrease of itching, redness and scaling of the skin therefore also leading to improved sleep and the ability to undertake social activities again (Table 2; Q21). For one patient the treatment was successful in the beginning however after several weeks the skin and blood results worsened despite the continuation of mogamulizumab.

The treatment with mogamulizumab required patients to visit the hospital every 2 weeks. For some patients this involves having to travel across the country for their treatment. Two patients mentioned the associated high financial costs of travel as the largest burden, whilst other patients discussed having to request help from others to assist them to the hospital appointments as the largest burden.

The number of reported side effects of mogamulizumab by patients during the interview was low. The most common side effect was fatigue in the day or days subsequent to the infusion, reported by all seven patients. Aside from the fatigue, one patient experienced a transfusion reaction and another patient a mogamulizumab‐associated rash. No participants had to discontinue treatment with mogamulizumab due to side effects.

### 3.3 Support

The significant impact of this disease resulted in support being another main theme. Most participants (6/7) received the greatest support from their family and/or partner, whilst one patient mentioned experiencing the most support from health professionals in the hospital. The diagnosis also led to strengthened family bonds and more appreciation by participants for their friends and family (Table 2; Q22). Family members were often also beneficial in examining the skin and by helping to apply ointments (Table 2; Q23). Although family members were the greatest source of support, three patients expressed facing difficulties sharing the diagnosis and discussing the consequences with their close friends and family. The main cause for this is that participants do not want to burden others and do not want to cause distress amongst family and friends. One patient reported that her faith was essential in giving her strength (Table 2; Q24).

Despite the lack of referral and/or diagnostic delay prior to the diagnosis, the majority of patients experienced sufficient support from health professionals after receiving the MF or SS diagnosis. In the health care process participants valued a short waiting period for appointments, clear and comprehensive patient‐education and a pleasant doctor‐patient relationship.

## 4 DISCUSSION

This study provides a broad insight into the QoL in patients with E‐CTCL receiving mogamulizumab. Exploration of the quality of life in erythrodermic MF/SS through semi‐structured interviews in this study led to five major themes: diagnosis, physical functioning, psychological and social functioning, treatment and support. To our knowledge, this study is the first qualitative assessment that studies the effect of mogamulizumab and the impact this treatment has on patients' QoL. Six out of seven participants subjectively reported an effective response to treatment with mogamulizumab with a decrease or resolution of physical symptoms, and improved social functioning, and overall QoL.

As found in previous quantitative and qualitative studies, we found that advanced‐stage CTCL has a significant impact on physical, psychological and social domains and greatly affects the QoL.^2^, ^12^ Diagnostic delay is a common problem in CTCL patients with six out of seven patients experiencing delay in referral or diagnosis as also shown in previous qualitative research.^9^ Three participants independently requested a second opinion which in all cases led to the CTCL diagnosis. This suggests that in the case of diagnostic uncertainty, timely referral is necessary. Prompt diagnosis is desired as studies have shown that delay may have a psychological impact and affect the management of symptoms.^13^ Receiving this diagnosis can have a great impact on patients and their families and may lead to disbelief and shock. However, some patients in our study reported that the diagnosis can also be seen as a relief of the uncertainty that patients experience prior to the diagnosis.

Pruritus was the most reported physical symptom in this study and has a large impact on the QoL, often leading to insomnia in case of extreme itching. An earlier study also portrays a direct correlation between itch and QoL while also reporting a higher prevalence of pruritus in advanced stage CTCL.^14^, ^15^ Adequate therapeutic management is needed to decrease the large burden that pruritus has on patients' QoL. The majority of participants in this study mentioned a significant decrease or complete resolution of pruritus after the start of the mogamulizumab infusions. Therefore, in accordance with previous literature, mogamulizumab appears to have a positive effect on itch severity and frequency.^8^, ^16^

The visibility of the E‐CTCL was another important theme which sometimes resulted in embarrassment, insecurities, and avoidance of social activities. This is enhanced by a lack of understanding and unsolicited remarks by strangers also became apparent in a previous QoL study, and is in line with previous research on the extent and impact of perceived stigmatisation in people with chronic visible skin conditions.^17^, ^18^ Mogamulizumab also seemed to have a positive effect on symptoms other than pruritus as participants reported a reduction in redness and scaling of the skin. This reduction led to an improvement in QoL as they were able to enjoy social activities without feelings of insecurity.

This study found that CTCL patients portray diverse coping mechanisms. Some show avoidance‐oriented coping strategies (avoiding social interaction, avoiding telling others about their diagnosis, and refraining from searching for information online), whilst others display adaptive strategies (e.g., finding it important to accept the illness and to seek support in their family and friends). Physicians should pay attention to a patients' illness perception and their coping mechanism, as studies have shown that recognition of maladaptive coping might improve adherence to treatment.^19^, ^20^ Additionally, adequate patient education is shown to improve symptom management by positively influencing patients' coping strategies.^21^

Our data suggests that the high symptom burden of CTCL seems to have a significant effect on the daily life of patients. Participants of this study reported that the disease had a large impact on their ability to work or to pursue their hobbies. The main cause for this seemed to be the fatigue and decreased physical stamina. Apart from the adjustments that had to be made in career and hobbies, patients also reported clothing adaptations. A previous study also reported that CTCL causes two thirds of patients to adapt their clothing choice.^4^

Whilst improving patients' QoL, few side effects were reported. The most common side‐effect were fatigue subsequent to the mogamulizumab infusion. Others included a drug rash and a transfusion reaction. This corresponds with an earlier study.^22^

Limitations of this study included a selection bias, that measurements were conducted at a single point of time and at various points of the mogamulizumab therapy in this single centre study. Treatment with mogamulizumab is often offered to patients with a high disease activity and/or symptom burden and might therefore not be completely representative of all advanced‐stage CTCL patients. The small sample size in our study should also be taken into account when generalising our findings even though data saturation was reached. We were not able to systematically study differences due to low sample size. To minimise bias we adhered to the qualitative research guidelines and reported our findings using the COREQ criteria.^10^, ^23^

## 5 CONCLUSION

E‐CTCL has major consequences on the physical functioning as well as on the emotional/psychological and social well‐being. Mogamulizumab appears to have positive effect on symptoms and improves patients' QoL on multiple domains. The findings from this study provide valuable information to health care professionals and guide them in improving their care strategy by management of/measuring pruritis, asking about the visibility of disease, their coping mechanisms, their support system, and provide realistic expectations.

## CONFLICT OF INTEREST STATEMENT

The authors declare no conflicts of interest.

## AUTHOR CONTRIBUTIONS

**Rosanne Ottevanger:** Conceptualisation (equal); data curation (equal); formal analysis (equal); investigation (equal); methodology (equal); project administration (equal); software (equal); writing – original draft (equal). **Judith S. Feenstra:** Conceptualisation (equal); data curation (equal); formal analysis (equal); investigation (equal); methodology (equal); project administration (equal); software (equal); writing – original draft (equal). **Liesbeth M. van Vliet:** Conceptualisation (equal); methodology (equal); writing – review and editing (equal). **Sylvia van Beugen:** Conceptualisation (equal); methodology (equal); writing – review and editing (equal). **Andrea W. M. Evers:** Conceptualisation (equal); methodology (equal); writing – review and editing (equal). **Cees Kennedy:** Conceptualisation (equal); methodology (equal); writing – review and editing (equal). **Rein Willemze:** Conceptualisation (equal); methodology (equal); supervision (equal); writing – review and editing (equal). **Maarten H. Vermeer:** Conceptualisation (equal); funding acquisition (equal); writing – review and editing (equal). **Koen D. Quint:** Conceptualisation (equal); methodology (equal); supervision (equal); writing – review and editing (equal).

## ETHICS STATEMENT

The patients in this manuscript have given written informed consent to publication of their case details. Although the sample size seems limited we achieved complete data saturation. Therefore, it was not ethical to further expand the cohort for this study.

## Data Availability

Data will be available upon reasonable request with the corresponding author.

## References

[1] Willemze R , Cerroni L , Kempf W , Berti E , Facchetti F , Swerdlow SH , et al. The 2018 update of the WHO‐EORTC classification for primary cutaneous lymphomas. Blood. 2019;133(16):1703–1714. 10.1182/blood-2018-11-881268 30635287 PMC6473500

[2] Ottevanger R , van Beugen S , Evers AWM , Willemze R , Vermeer MH , Quint KD . Quality of life in patients with Mycosis Fungoides and Sezary Syndrome: a systematic review of the literature. J Eur Acad Dermatol Venereol. 2021;35(12):2377–2387. 10.1111/jdv.17570 34331819 PMC9291074

[3] Scarisbrick JJ , Prince HM , Vermeer MH , Quaglino P , Horwitz S , Porcu P , et al. Cutaneous Lymphoma International Consortium study of outcome in advanced stages of mycosis fungoides and Sezary syndrome: effect of specific prognostic markers on survival and development of a prognostic model. J Clin Oncol. 2015;33(32):3766–3773. 10.1200/jco.2015.61.7142 26438120 PMC4979132

[4] Demierre MF , Gan S , Jones J , Miller DR . Significant impact of cutaneous T‐cell lymphoma on patients' quality of life: results of a 2005 National Cutaneous Lymphoma Foundation Survey. Cancer. 2006;107(10):2504–2511. 10.1002/cncr.22252 17048251

[5] Willemze R , Jaffe ES , Burg G , Cerroni L , Berti E , Swerdlow SH , et al. WHO‐EORTC classification for cutaneous lymphomas. Blood. 2005;105(10):3768–3785. 10.1182/blood-2004-09-3502 15692063

[6] Kasamon YL , Chen H , de Claro RA , Nie L , Ye J , Blumenthal GM , et al. FDA approval summary: mogamulizumab‐kpkc for mycosis fungoides and Sezary syndrome. Clin Cancer Res. 2019;25(24):7275–7280. 10.1158/1078-0432.ccr-19-2030 31366601

[7] Porcu P , Hudgens S , Horwitz S , Quaglino P , Cowan R , Geskin L , et al. Quality of life effect of the anti‐CCR4 monoclonal antibody mogamulizumab versus vorinostat in patients with cutaneous T‐cell lymphoma. Clin Lymphoma Myeloma Leuk. 2021;21(2):97–105. 10.1016/j.clml.2020.09.003 33158772 PMC7878351

[8] Ottevanger R , van Beugen S , Evers AWM , Willemze R , Vermeer MH , Quint KD . Quality of life in cutaneous T‐cell lymphoma patients receiving mogamulizumab: important factors to consider. Cancers. 2022;15(1):32. 10.3390/cancers15010032 36612028 PMC9817675

[9] Beynon T , Selman L , Radcliffe E , Whittaker S , Child F , Orlowska D , et al. 'We had to change to single beds because I itch in the night': a qualitative study of the experiences, attitudes and approaches to coping of patients with cutaneous T‐cell lymphoma. Br J Dermatol. 2015;173(1):83–92. 10.1111/bjd.13732 25688924

[10] Tong A , Sainsbury P , Craig J . Consolidated criteria for reporting qualitative research (COREQ): a 32‐item checklist for interviews and focus groups. Int J Qual Health Care. 2007;19(6):349–357. 10.1093/intqhc/mzm042 17872937

[11] Braun V , Clarke V . Using thematic analysis in psychology. Qual Res Psychol. 2006;3(2):77–101. 10.1191/1478088706qp063oa

[12] Demierre MF , Tien A , Miller D . Health‐related quality‐of‐life assessment in patients with cutaneous T‐cell lymphoma. Arch Dermatol. 2005;141(3):325–330. 10.1001/archderm.141.3.325 15781673

[13] Barke J . Qualitative accounts of living with cutaneous T‐cell lymphoma. Br J Dermatol. 2015;173(1):10–11. 10.1111/bjd.13841 26174639

[14] Serrano L , Martinez‐Escala ME , Zhou XA , Guitart J . Pruritus in cutaneous T‐cell lymphoma and its management. Dermatol Clin. 2018;36(3):245–258. 10.1016/j.det.2018.02.011 29929596

[15] Ottevanger R , van Beugen S , Evers AW , Willemze R , Vermeer MH , Quint KD . Itch in patients with Cutaneous T cell lymphoma as a quality of life indicator. JAAD Int. 2022;9:57–64. 10.1016/j.jdin.2022.07.007 36147217 PMC9486110

[16] Kim YH , Bagot M , Pinter‐Brown L , Rook AH , Porcu P , Horwitz SM , et al. Mogamulizumab versus vorinostat in previously treated cutaneous T‐cell lymphoma (MAVORIC): an international, open‐label, randomised, controlled phase 3 trial. Lancet Oncol. 2018;19(9):1192–1204. 10.1016/s1470-2045(18)30379-6 30100375

[17] Bhat TS , Herbosa CM , Rosenberg AR , Sogade O , Jeffe DB , Mehta‐Shah N , et al. Current measures are not sufficient: an interview‐based qualitative assessment of quality of life in cutaneous T‐cell lymphoma. Br J Dermatol. 2021;184(2):310–318. 10.1111/bjd.19298 32510571 PMC7722174

[18] Luck‐Sikorski C , Rossmann P , Topp J , Augustin M , Sommer R , Weinberger NA . Assessment of stigma related to visible skin diseases: a systematic review and evaluation of patient‐reported outcome measures. J Eur Acad Dermatol Venereol. 2022;36(4):499–525. 10.1111/jdv.17833 34817889

[19] Porkert S , Lehner‐Baumgartner E , Valencak J , Knobler R , Riedl E , Jonak C . Patients' illness perception as a tool to improve individual disease management in primary cutaneous lymphomas. Acta Derm Venereol. 2018;98(2):240–245. 10.2340/00015555-2819 29048099

[20] Wahl AK , Robinson HS , LANgELAND E , Larsen MH , Polesie ALK , Moum TÅ . Clinical characteristics associated with illness perception in psoriasis. Acta Derm Venereol. 2014;94(3):271–275. 10.2340/00015555-1673 24002676

[21] Garchinski CM , DiBiase A.‐M , Wong RK , Sagar SM . Patient‐centered care in cancer treatment programs: the future of integrative oncology through psychoeducation. Future Oncol. 2014;10(16):2603–2614. 10.2217/fon.14.186 25531048

[22] Lewis DJ , Rook AH . Mogamulizumab in the treatment of advanced mycosis fungoides and Sezary syndrome: safety and efficacy. Expert Rev Anticancer Ther. 2020;20(6):447–452. 10.1080/14737140.2020.1760096 32320304

[23] Malterud K . Qualitative research: standards, challenges, and guidelines. Lancet. 2001;358(9280):483–488. 10.1016/s0140-6736(01)05627-6 11513933

